# Altering undigested neutral detergent fiber through additives applied in corn, whole barley crop, and alfalfa silages, and its effect on performance of lactating Holstein dairy cows

**DOI:** 10.5713/ajas.18.0314

**Published:** 2019-02-20

**Authors:** Seyed Mohsen Hosseini, Mohsen Danesh Mesgaran, Ali Reza Vakili, Abbas Ali Naserian, Ehsan Khafipour

**Affiliations:** 1 Department of Animal Science, Faculty of Agriculture, Ferdowsi University of Mashhad, P.O. Box 91775-1163, Mashhad, Iran; 2 Department of Animal Science, University of Manitoba, Winnipeg, Manitoba, R3T 2N2, Canada

**Keywords:** Corn, Whole Barley Crop, Alfalfa, Undigested Neutral Detergent Fiber, Milk, Cow

## Abstract

**Objective:**

We hypothesized that silage additives may alter the undigested neutral detergent fiber (uNDF) content through ensiling. Therefore, urea and formic acid were applied to corn, whole barley crop (WBC) and alfalfa to change uNDF content of the ensiled forages.

**Methods:**

Six experimental diets at two groups of high uNDF (untreated corn and alfalfa silages [CSAS] and untreated whole barley and alfalfa silages [BSAS]) and low uNDF (urea-treated corn silage+untreated alfalfa silage [CS_U_AS], urea-treated whole barley silage+untreated alfalfa silage [BS_U_AS], untreated corn silage+formic acid-treated alfalfa silage [CSAS_F_], and untreated whole barley silage+formic acid-treated alfalfa silage [BSAS_F_]), were allocated to thirty-six multiparous lactating Holstein dairy cows.

**Results:**

The untreated silages were higher in uNDF than additive treated silages, but the uNDF concentrations among silages were variable (corn silage<barley silage<alfalfa silage). Dry matter intake was not influenced by the reduction of uNDF or physical NDF supply source from corn to WBC silages (p>0.05). Milk yield tended to increase in the cows fed high uNDF diets than those fed low uNDF (p = 0.10). The cows fed diet based on urea-treated corn silage had higher milk yield than those fed other silages (p = 0.05). The substitution of corn silage with the WBC silage tended to decrease milk production (p = 0.07). Changing the physical source of NDF supply and the uNDF content from the corn silage to the WBC silage caused a significant increase in ruminal NH_3_-N concentration, milk urea-N and fat yield (p< 0.05). The cows fed diets based on WBC silage experienced greater rumination time than the cows fed corn silage (p<0.05).

**Conclusion:**

Administering additives to silages to reduce uNDF may improve the performance of Holstein dairy cows.

## INTRODUCTION

Silages have become the necessary forage component in the ration of dairy cows over the last few decades [1]. They are often a wise choice when selecting forages for providing fiber and energy needed to optimize rumen function [2]. A critical component for assessing of silage quality is fiber digestibility that impact on feed intake and milk yield [2]. Silage digestibility, which is a complex function, is related to the dynamic processes of degradation and passage rate from the rumen [3]. A 0.01 unit increase in neutral detergent fiber (NDF) degradability cause an increase in daily intake of 0.17 kg and fat-corrected milk (FCM) with 0.25 kg/d [4]. Raffrenato and Van Amburgh [5] noted that fiber digestibility relates to two digestible pools, followed the first order kinetic, but each has a different rate of digestion; fast digestion was defined at 24 to 30 h and slow digestion at 96 to 120 h. Undigested NDF (uNDF) defined as the functional fiber fraction that influences physical effectiveness, gut fill, potential microbial protein from digested NDF in the rumen, and digestion/passage dynamics of forage samples [6]. The uNDF of a feed is a better analytical indicator of nutritional availability than either NDF digestibility or NDF because uNDF can be used to predict potentially digestible NDF (pdNDF; defined as NDF - uNDF), estimated the NDF pools and rates of fiber fermentation, influenced dry matter intake (DMI) and chewing response. Therefore, any process in the silage that changes NDF digestion or uNDF content may affect the performance of an animal [7]. Silage additives have been used to enhance the ensiling fermentation by preventing the production of undesired acids to produce well-preserved silages [8]. Urea is a synthetic, non-protein nitrogen compound classified as a nutrient silage additive, because it is a source of nitrogen for bacteria in the rumen [9]. It seemed that corn silage (a high energy, low protein feed) might be an ideal type of feed to be considered for use with urea as an additive to increase its crude protein content. Formic acid, as an inhibitor of fermentation and for its antibacterial effect is an organic acid cause a reduction in protein degradation and deamination in legume such as alfalfa [10] and improves animal performance [11,9]. Cushnahan and Mayne [12] showed no effect of restricting silage fermentation on NDF digestibility. In contrast, Weimer [7] suggested that the buffering effect of silage additive on rumen pH might be a possible explanation for the beneficial effects on fiber digestibility. However, there has been limited literature or research on the evaluation of silage based on fiber digestion and uNDF. In this study, we hypothesized that i) Inclusion of additives during ensiling improve fermentation characteristics, ii) Additives influence fiber digestion and decrease uNDF by changing silage chemical properties followed by increasing milk yield and milk composition, and iii) Corn and whole barley crop (WBC) silages have different responses to performance and chewing activity owing to various intrinsic uNDF and physical breakdown. Therefore, the aim of the present study was first to evaluate the effects of urea and formic acid applied in corn, WBC and alfalfa silages in an effort to alter uNDF using *in vitro* incubation with buffered rumen fluid. Then, to investigate the effect of uNDF in lactating Holstein dairy cow diets on performance, ruminal characteristics, chewing activity and selected blood metabolites.

## MATERIALS AND METHODS

### Silages management, animals and feeds

The corn forage (hybrid 700) on 17 September 2016 at 2/3 milk line of kernel maturity stage, WBC (*Hordeum vulgare* L.) on 20 April 2016 at dough stage, and alfalfa forage (*Medicago sativa* L.) on 10 May 2016 in second cutting at 40% flowering from a single field were harvested and chopped using a pull-type chopper (model 965, Claas, Omaha, NE, USA). Then, they were assigned to two groups of untreated and treated with urea at 21.6 g/kg based on dry matter (DM) for corn and whole barley and formic acid at 4.4 L/ton of fresh alfalfa (10% higher of founding Nagel and Broderick [13]) and ensiled for 40 days in trench silos (approximately 10 ton per each silo), sealed with two layers of plastic sheeting. Silage samples were individually evaluated using *in vitro* incubation [14] to determine undigested NDF (uNDF). The uNDF was evaluated at intervals of 30 (fast pool), 120 (slow pool), and 240 hours (uNDF_30h_, uNDF_120h_, and uNDF_240h_, respectively). Buffered rumen fluid preparation and procedure have been fully described by Raffrenato and Van Amburgh [5]. Briefly, rumen fluid was obtained from two rumen fistulated Holstein steers (310±11 kg body weight [BW], 11±0.3 month age) which fed 2.1 kg of DM alfalfa hay, 3.2 kg of DM corn silage and 2.5 kg of DM concentrate plus supplemental vitamins and minerals (158 g crude protein [CP]/kg of DM). Rumen fluid and digesta were mixed with a blender, clarified through four layers of cheesecloth and strained through a nylon fabric with 46 μm pore size. Exactly 500 mg of ground silage samples were placed into a 120 mL glass bottle and then, 40 mL buffer accompanied by 10 mL of rumen fluid was added to each in triplicate with three runs. The samples that fermented longer than 120 hours were re-inoculated with the same amount of the initial rumen liquor/medium mix, as mentioned in Raffrenato and Van Amburgh [5]. At the end of fermentation, according to the specific time intervals, residue was analyzed to determine NDF content according to Van Soest et al [15]. The silages were included in the experimental diets (n = 6) to achieve high and low uNDF concentrations. Therefore, in high uNDF diets the untreated corn and alfalfa silages (CSAS) and untreated whole barley and alfalfa silages (BSAS) were used. While in low uNDF diets we used urea-treated corn silage+ untreated alfalfa silage (CS_U_AS), urea-treated whole barley silage+untreated alfalfa silage (BS_U_AS), untreated corn silage+ formic acid-treated alfalfa silage (CSAS_F_) and untreated whole barley silage+formic acid-treated alfalfa silage (BSAS_F_). All diets were formulated iso-nitrogenous and iso-energetic to meet all nutritional requirements of the dairy cows as described by the National Research Council ([16]; Table 1). All concentrates and alfalfa hay put aside to ensure all diet ingredient used in ration was a similar nutritional content thorough of trial. The pen state particle size separator was used for determined silage theoretical cutting length (Table 2; [17]). Thirty-six multiparous Holstein dairy cows in mid-lactation (590±7 kg BW; means±standard deviation) averaging 200±14 days in milk and producing 29±4 kg/d of milk were allocated into six groups (each group contained 6 cows) thorough of this trial. Experimental period lasted 56 days with the first 14 days pre-treatment for adaptation period followed by 42 days for sampling. Animals were cared and the Iranian Institutional Animal Care Committee [18] approved the experiment for animals used in research. The cows were housed in a tie stall barn covered by a roof in individual pens measuring 1.10×2.50 and each cow stayed in its own pen thorough of the trial. The cows had free access to water and were fed *ad libitum* and received feed second times a day at 0800 and 1600 directly after milking. The feed allocated was increased or decreased with feed intake, until a 10% residue was achieved. The cows were allowed 10 min of exercise, three times daily.

### Recordings and sampling

After opening the plastic silos, four representative samples of the fresh silages of corn, WBC and alfalfa were collected from different part for determination of pH, NH_3_-N concentration and chemical composition. In sampling period, DMI was determined as the difference between total mixed ration (TMR) offered and orts weighed daily, then samples of orts collected and pooled per cow for the determination of chemical composition. The cows were weighed at the beginning and the end of the experimental period. The milk production was electronically recorded daily, at each individual milking, through the experiment. Milk samples were taken every week and sent to the Dairy Laboratory for the determination of milk composition. Milk composition was calculated as an average of morning and afternoon samples using the proportion of daily production at that milking as a weighting factor. Blood samples were collected at 07:00 am before the morning feeding from the jugular vein into two evacuated serum tubes containing clot activator (BD Vacationer Systems; Becton, Dickinson and Company, Franklin Lakes, NJ, USA). All blood samples were transported to the lab in an ice bucket, and serum was separated by centrifugation at 1,600×g and 4°C for 15 min, and then stored at −20°C until assaying of glucose, total cholesterol, alanine aminotransferase, aspartate aminotransferase (AST), and blood urea-N. The fecal samples were collected directly from the rectum once daily at noon on the last five consecutive days and pooled per cow. Then they were stored at −20°C, until chemical analysis. Apparent total tract digestibility coefficients were determined by acid insoluble ash technique (AIA) as an internal marker [19,20]. At the end of period, rumination activity was monitored for every cow over a 24 h visually (one expert person was considered for every 12 cows). Eating, ruminating and water consumption activities were recorded at 5-min intervals, and each activity was assumed to persist during the entire 5 min interval [21]. A body condition score was performed during afternoon milking. A score 1 to 5 was given to each cow, one indicating severe under nutrition and five indicates sever obesity [22]. Body surface temperatures were collected in order to assess differences in body temperature among cows at the end of experimental period. Body surface temperatures of each cow recorded using a thermal imaging camera (Fluke Ti25 Infrared Camera; Fluke Corporation, Everett, WA, USA). At the end of period, 4 h after the morning feeding, the rumen fluid was sampled for the determination of pH value, NH_3_-N and volatile fatty acids (VFA) concentrations. The pH value of the ruminal fluid was immediately determined using digital portable pH meter (WinLab, portable) and samples were immediately frozen at −20°C. For NH_3_-N analysis, 20 mL of the filtered rumen fluid was transferred to tubes and preserved with 20 mL of a 0.1 N sulphuric acid solution [23]. For VFA analysis, 20 mL of the filtered rumen fluid was transferred to second tube and 5 mL of a 25% meta-phosphoric acid solution was added to preserve the sample.

### Chemical analysis

Silage and concentrate samples were analyzed for CP (N×6.25; AOAC [24]; method 990.03, using Kjeltec2300 Auto analyzer, Foss Tecator AB, Hoganas, Sweden), NDF (assayed without heat stable amylase and sodium sulphite) and acid detergent fiber (ADF; Van Soest et al [15]), and also buffering capacity for silage samples (BC [25]). Moreover, CP, NDF, and DM contents were determined for AIA analysis for both feed and fecal samples. Water soluble carbohydrate (WSC [26]) concentration were measured for silages by an anthrone-sulphuric acid procedure using glucose as standard. Aerobic stability was defined as the time needed the silage remained stable before increasing to more than 2°C above the ambient temperature and measured in accordance with methodology given by Kleinschmit and Kung [27]. Samples of silage extract for pH value and NH_3_-N concentration were prepared by blending 50 g of fresh silage and 450 mL of doubled distilled water (w/v) to a homogenized state using a blender for 2 min, then pH was determined immediately by a digital portable pH meter (WinLab, portable). A portion of extracts strained through 4 layers of cheesecloth and 5 mL of fluid samples were acidified with 5 mL of 0.2 N HCL, then centrifuged at 3,500×g for 10 min, to determine NH_3_-N concentration. The rumen VFA composition was determined by gas chromatography (Chrompack, model CP-9002; Chrompack International BV, Middelburg, the Netherlands) using a 50-m fused-silica column (CP-Wax Chrompack Capillary Column; Varian Inc., Palo Alto, CA, USA), and crotonic acid as the internal standard. Nitrogen was used as carrier gas, and oven initial and final temperatures were 55°C and 195°C, respectively. Detector and injector temperatures were set at 250°C.

### Calcluations

The uNDF was calculated as 100 – *in vitro* NDF digestibility (IVNDFD), where IVNDFD was % NDF = (1–[NDF_residual_–NDF_blank_]/NDF_sample_)×100, the amount of pdNDF is calculated from the difference of total NDF and uNDF (pdNDF as % DM = [100–% uNDF_240 h_]×% NDF_sample_; [28]). The yield of 3.5% FCM and energy-corrected milk (ECM) were calculated according to NRC equations [16]:

$$\begin{array}{l} \text{FCM}=0.432\times \text{milk yield}+16.23\times \text{fat yield}\\ \text{ECM}=12.82\times \text{fat yield}+7.13\times \text{protein yield}+0.323\times \text{milk yield} \end{array}$$

Apparent digestibility coefficients (%) for DM, CP, and NDF were calculated as: ([1–{F/D}×D_m_/F_m_])×100 Where F = % nutrient in faeces, D = % nutrient in diet, D_m_ = % marker in diet, and F_m_ = % marker in faeces.

### Statistical analysis

In order to assess the differences between non-repeated data including silage composition, blood metabolites, pH value, rumen fermentation, body temperature and total tract digestibility, they were analyzed using the general linear models procedure of SAS [29]. Statistical model was considered: Y_ij_ = μ+T_i_+ɛ_ij_, where Y_ij_ is the response for the jth observation of the ith treatment, μ is the population mean, T_i_ is the ith treatment effect and ɛ_ij_ is the random residual. Data of feed intake, milk production and composition as well as body score, were analyzed using mixed model as repeated measure procedure of SAS (SAS Institute, Cary, NC, USA). Treatment considered as fixed effect. The REML method was used to estimate least squares means (LSM), and the Kenward-Roger method was used to calculate denominator degrees of freedom. The variance-covariance error structure was compound symmetry, because it gave minimum akaike information and corrected bayesian information criterions. The statistical model used for analyses was: Y_ij_ = μ+T_i_+A_j_+ɛ_ij_, where Y_ij_ was each observation, μ was the overall mean, T_i_ was the fixed effect of treatment i, A_j_ was the random effect of cow and ɛ_ij_ was residual error. The interaction between treatment and time was also included in the model, but it was not significant and dropped from the model. Contrasts were tested using the CONTRAST statement of SAS to evaluate the high and low uNDF diets and also the corn silage compared with the WBC silage. Differences among means were tested using the LSMEANS test. The PDIFF option in the LSMEANS statement was used to separate means. Standard errors of means were calculated from the residual mean square in analysis of variance. Significance was declared at p≤0.05 and tendencies were explained at p≤0.10.

## RESULTS

### Silage chemical composition and quality evaluation

The type of silage (corn, WBC, and alfalfa), regardless of the additives used, affected the concentrations of DM, ADF, and WSC, and the buffering capacity (mENaOH/100 g DM; BC) (Table 3). Accordingly, BC and ADF concentration were the highest for the alfalfa silage, intermediate for the WBC silage, and the lowest for the corn silage. The DM and WSC concentrations were the highest for the corn and WBC silages, respectively, while the alfalfa silage had the lowest DM and WSC concentrations. Ammonia-N concentrations and crude protein were increased by supplementation urea to the corn and WBC silages (p<0.05). Formic acid-treated alfalfa silages had lower NH_3_-N concentration and higher protein content than those of the untreated silages (p<0.05). Urea improved aerobic stability in the corn and WBC silages. Although the alfalfa silage had higher aerobic stability than those of the corn and WBC silages (p<0.05), formic acid unaffected aerobic stability (p>0.05). The undigested NDF at 30 and 120 h of incubation was affected by urea in the corn and WBC silages; it was lower than those of the untreated silages (p<0.05). However, formic acid decreased only uNDF at 30 h incubation. We observed no detectable differences for uNDF at 240 h across additives and their untreated silages, although it was affected by type of silages and was the highest for the alfalfa, intermediate for the WBC and the lowest for the corn silages.

### Feed intake, digestibility and animal body status

The DMI, final BW, BW changes thorough the trail and means of body condition scores were similar across cows fed high or low uNDF diets and also between two different NDF sources of corn and WBC silages (Table 4). The daily silage uNDF_30h_ and uNDF_120h_ intake were lower in the cows fed diets based on urea and formic acid treated silages than those fed the untreated silages (p<0.05). The daily silage uNDF_240h_ intake was lower in the CS_U_AS and BS_U_AS groups than those of the CSAS and BSAS (p<0.05), while uNDF_240h_, unlike uNDF_30h_ was same in the cows fed the CSAS_F_ and BSAS_F_ compared with the CSAS and BSAS groups. Additionally, the cows fed diets based on corn silage compared with those received the WBC silage had much lower the daily uNDF intake (p<0.05). Body surface temperatures were similar among the treatments (p> 0.05). Feed efficiency was the highest in the CS_U_AS than those of the other treatments (p<0.05). The apparent total tract digestibility of NDF (NDFD) was higher in the cows fed low uNDF silages than those fed the high uNDF, untreated silages (p<0.05). However, the apparent total tract digestibility of dry matter and crude protein were not affected among the cows fed high or low uNDF (p>0.05). Likewise, we observed no significant differences between diets based on the corn and WBC silages for total tract digestibility of the nutrients.

### Milk yield and composition

**Figure 1:**
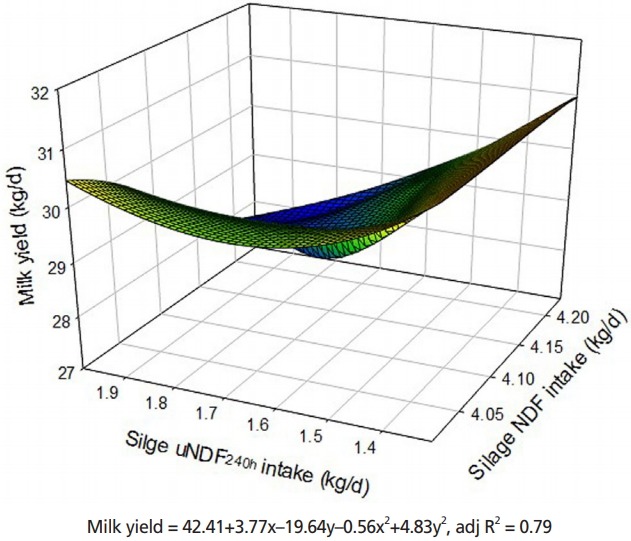
3D surface responses to silage NDF and uNDF_240h_ intake on milk yield of Holstein dairy cows. NDF, neutral detergent fiber; uNDF, undigested NDF.

The data related to milk yield and milk composition as a result of the different uNDF rations are presented in Table 5. Decreasing uNDF due to the inclusion of urea to the corn silage (CS_U_AS) increased milk yield, while the addition of formic acid to the alfalfa (CSAS_F_ and BSAS_F_) did not change milk production. Compared to diets based on the WBC silage, the corn silage tended to increase (p = 0.07) milk production. When the intake of silage NDF and uNDF increased, the milk production decreased and was minimum when the cows were fed diets based on silages with 4.15 and 1.99 kg of NDF and uNDF, respectively (Figure 1; adjR^2^ = 0.79). Fat-corrected milk and energy-corrected milk yields were unaffected by the uNDF levels or the source of NDF supplied in the diets (p> 0.05). Although reducing uNDF through the inclusion of additives did not affect milk fat yield and percentage, the change of NDF source from the corn silage to the WBC silage caused a significant increase in fat yield and fat percentage (p<0.05). Milk urea-N was higher in the cows fed CS_U_AS and BS_U_AS than those fed CSAS, CSAS_F_, BSAS and BSAS_F_ (p<0.05). Replacing the source of NDF, as a result of changing from the corn silage to the WBC silage, significantly increased milk urea-N (p<0.05). Solid non-fat milk (SNF) was the highest in the cows fed the CS_U_AS and the lowest in those fed the BSAS_F_. The substitution of corn silage with WBC silage decreased the SNF content. The effects of the different uNDF levels or NDF sources were not observed on protein, lactose, and total solid across the treatments (p>0.05).

### Ruminal fermentation characteristics

Ruminal propionate, acetate and butyrate concentrations as well as acetate to propionate ratio were not affected by uNDF levels in the cows fed the experimental diets (p>0.05; Table 6). The type of physical NDF included in the experimental ration did not change the VFA concentrations in the cows fed diets based on the corn silage rather than those fed the WBC silage. Moreover, we observed no significant differences for ruminal pH value across the treatments with various uNDF (p>0.05). Reducing uNDF in the CS_U_AS and BS_U_AS caused higher NH_3_-N concentration in the rumen. Nevertheless, the CSAS_F_ had lower NH_3_-N concentration than those fed the CSAS (p<0.05). Furthermore, the cows fed diets based on corn silage experienced lower NH_3_-N concentration than those fed the WBC silage (p<0.05).

### Eating and ruminating

**Figure 2:**
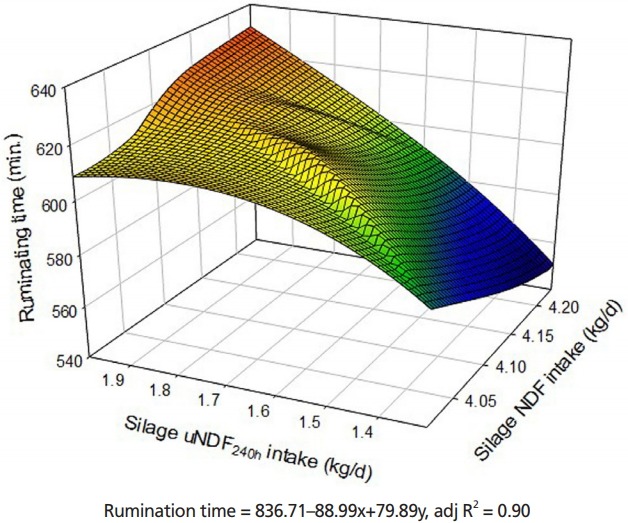
3D surface responses to silage NDF and uNDF_240h_ intake on ruminating time of Holstein dairy cows. NDF, neutral detergent fiber; uNDF, undigested NDF.

The results of eating, ruminating and water drinking activities are reported in Table 7. There were no significant differences in eating time (min/d) and eating time adjusted for DM and NDF intake (min/kg of DM and NDF, respectively) among the diets based on high or low uNDF content and also, for sources of NDF supply through the corn or WBC silages (p> 0.05). Although rumination time was unaffected by reducing uNDF via inclusion of urea or formic acid to the corn, WBC and alfalfa silages, the cows fed diets based on WBC silage experienced greater ruminating time than those fed the corn silage, as different uNDF content and physical NDF supplied source (p<0.05). Longer ruminating time associated with substitution of the corn silage with the WBC silage was not corresponded to eating time. Hence, total chewing activity (eating+ruminating) was not affected by the type of silages (p>0.05). There was a strong relationship between silage NDF and uNDF intake with rumination time and the highest rumination activity was observed when the cows were fed diets based on silages with 4.1 kg NDF and 1.99 kg uNDF (Figure 2, adjR^2^ = 90). There were no detectable differences for water drinking time (min/d) among cows fed different levels of uNDF (p>0.05). Nevertheless, the cows fed diets based on the WBC silage tended to have longer times of water drinking adjusted for DM and NDF (p = 0.10) compared with those fed the corn silage.

### Blood metabolites

Decreasing uNDF due to the inclusion of urea and formic acid to the silages did not affect the concentrations of glucose, triglyceride, cholesterol, and AST (Table 8). However, blood urea-N and alanine aminotransferase concentrations increased in the cows fed low uNDF diet (CS_U_AS and BS_U_AS; p<0.10). The substitution of WBC silages with corn silage increased blood urea-N (p = 0.03) as well as alanine aminotransferase concentrations (p = 0.09). Nonetheless, no significant differences for blood concentrations of glucose, triglyceride and AST for two different source of uNDF (corn vs WBC) were observed (p>0.05).

## DISCUSSION

### Silage fermentation and animal performance

In the present study, we evaluate the effect of various organic additives, which are mostly used in commercial dairy farms, on the pattern of the corn, WBC and alfalfa silages fermentation. In addition, we hypothesis whether the additives may alter the nature of NDF during ensiling. It has previously proposed that the silage additives can affect nutritional value and fermentation characteristics of the ensiled forages [30,9]. Therefore, we speculate that the treated silages lead to changes in uNDF. Therefore, the treated silages in dairy diets may impact the feed intake, nutrient digestibility, chewing activity, milk yield, and milk composition of lactating Holstein dairy cows.

Throughout the experiment, the urea-treated corn and WBC silages had higher CP and NH_3_-N concentrations and higher pH value as well as aerobic stability. However, NDF, uNDF at 30 and 120 h decreased by applying urea to the corn and WBC silages compared with the untreated silages. These findings do confirm previous findings which indicate that urea addition canimprove aerobic stability during feeding [31] and increase pH and ammonia-N concentration [32]. Urea is transformed into ammonia during ensiling. The antimitotic effect of ammonia decreased fungal growth because of the alkaline environment, so urea treated silages had higher aerobic stability [9]. The higher CP and lower ammonia-N concentration in the formic acid-treated silages were expected because acids cause a reduction in protein degradation. These results are in line with Jaakkola et al [10] who state that the addition of formic acid to silages may increase protein content and reduce ammonia-N concentration. The higher pH in the WBC and alfalfa silages compared with the corn may be due to their higher buffering capacity (BC), as the higher CP content of the alfalfa and WBC forages would increase the BC [8]. Greater WSC concentration in the WBC silage than the corn silage is in agreement with Addah et al [33] who noted that WSC concentration of whole barley forage ensiled was 5 times higher than those of corn.

In the present study, six diets, with the treated and untreated silages, were used. The diets were assigned by different types of silages and uNDF concentrations (low vs high). The results obtained from the lactating Holstein dairy cows demonstrated that DMI was unaffected by the daily silage uNDF intake. This suggests that ruminal pdNDF rate of digestion may drive DMI rather than uNDF. In the study of Grant and Cotanch [34], who used diets based on high or low forage (conventional vs brown midrib corn silage) with different fiber digestibility and uNDF, it was reported that the filling effect of the diet could be related to the amount and rate of degradation of pdNDF fraction rather than uNDF. The lack of impact of replacing the corn silage with the WBC silage in the cows’ diet on DMI was not expected. Indeed, because the amount of uNDF at 30 h, 120 h, and 240 h was higher in the WBC silage than that of the corn silage (Table 3), we expected a reduction in DMI as a result of feeding the WBC silage as compared to the corn silage. There was no significant difference among the dietary treatments for body surface temperature; results are expected because of lack of differences in acetate and acetate: propionate ratio (Table 6). A difference in body temperature may reflect a change in the VFA concentration. When acetate in the rumen increased, an increment body temperature occurred due to the heat of fermentation associated with acetate production [35].

In the present study, unlike DMI, the milk yield was improved by reducing the daily uNDF intake due to the inclusion of urea in the corn silage. These findings do confirm the research of Huber and Thomas [36], who reported the incorporation of urea into dairy cattle rations promotes milk production. However, distinct from their experiment, our rations were iso-nitrogenouse while differences for crude protein content among their dietary experiment affected DMI and consequently, milk yield. Furthermore, in our study, the increase in the milk yield of cows fed the CA_U_AS might be due to the reduction of uNDF via urea during silage fermentation. During ensiling, the partial hydrolase of NDF with additives were occurred (Table 3), having an impact on total tract NDF digestibility (Table 4). Therefore, this pre-hydrolase in silages caused to alter readily NDF digestion in the rumen by increasing surface area available for microbial attack, resulting in a more rapid rate of ruminal fermentation and increased milk yield. Another reason behind the increased milk yield in cows fed the CS_U_AS than those which take the CSAS might be due to higher silage pH with the inclusion of urea, followed by its buffering effects on fiber digestibility [7] (Table 3). Likewise, Shaver et al [37] reported that the silage pH is a factor that affects voluntary feed intake and organic matter intake increased when corn silage pH is elevated. The silage types (WBC vs corn) affected the milk yield. The milk production was higher in cows fed diets based on the corn silage than those fed the WBS (30.11 vs 28.66 kg/d/head). In accordance with our results, previous works report a greater milk yield in cows fed corn silage as opposed to cows fed barley silage [38]. However, a few recent studies concluded with a similar effect of diets based on corn or barley silages on milk yield [39]. Decreasing silage NDF and uNDF intake caused an increase in the milk yield and the maximum milk yield was allocated to the cows fed diet based on silages containing 4.0 and 1.3 kg/d of NDF and uNDF_240h_, respectively (Figure 1). These measurements were 0.7% and 0.22% of BW for NDF and uNDF_240h_, respectively. Mertens [40] reported that maximum NDF intake was 1.47% BW and forage NDF intake was 1.05% BW. He also noted that the range of undigested NDF (uNDF_240h_) should not be more than 0.30% to 0.48% BW [40]. Increased milk fat yield and fat percentage with changing NDF source from the corn silage to the WBC silage (1.21 and 4.05 vs 1.27 and 4.39 kg/d/head, respectively) suggests a longer chewing time for cows fed the WBC silages because of physical properties of fiber and the highest uNDF content. The greater milk urea-N in the cows fed diets based on WBC silage than those fed corn silage (18.64 vs 15.53, kg/d/head) were majority attributable to the fact that cows fed the WBC silage had higher NH_3_-N concentration in the rumen, which likely reflect to nitrogen construction and utilization between the corn and WBC silages. Another possibility could be that corn silage provides more energy for rumen microbes since it has low uNDF at 30, 120, and 240 h incubation (Table 3).

The similar pH, VFA concentration, and acetate: propionate ratio between low and high uNDF diets and the source of silage is partly attributable to the fact that the DMI across the treatments was the same (Table 6). Decreasing the pH and increasing the VFA concentration may decrease DMI [41], but this was not observed in our study. One possible explanation for having same pH value might be traced to the fact that in our study, the rations contained higher forage and, consequently, the rumen environment was favorable among treatments. The lower rumen NH_3_-N concentration in the diet of cows fed on meals that contained CSAS_F_ and BSAS_F_ in comparison to CSAS and BSAS might have led to a decrease in milk urea-N.

### Rumination and blood metabolites

The greater ruminating activity for the cows fed diets based on WBC silage than corn silage (318.88 vs 283.33, kg/d/head) might be related to the uNDF intake and physical effective NDF properties between two types of silages. The chewing time (min/d) of cows fed on CS_U_AS and BS_U_AS was no different in comparison to those fed on CSAS and BSAS, which reflects a lack of significant differences in feed intake (Table 4). Kononoff et al [42] also reported that DMI is an important driver of rumination time. Results of our study indicate that greater water drinking time adjusted per unit of DM and NDF in the cows fed diets based on WBC silage was higher than those fed corn silage might be consisted with initial DM content of silages. The WBC silage had lower DM content than corn silage (Table 3); consequently, the cows fed diets based on the WBC silage spent more time on drinking water to compensate in comparison to those fed corn silage. Ruminating time increased with increasing silage NDF and uNDF_240h_ intake (Figure 2), which confirms the results of Beauchemin and Buchanan-Smith [43] and Schulze et al [44], who reported that when the NDF concentration in the rations increase, cows and heifers spent more time on chewing. Low uNDF diets based on the CS_U_AS and BS_U_AS contained more non-protein nitrogen than untreated high uNDF diets (CSAS and BSAS). This may explain the observations of higher rumen NH_3_-N and the corresponding increases in blood urea-N. Screening alanine aminotransferase in serum is as an indicator for liver function and metabolite status [45]. Increasing alanine aminotransferase in the cows fed diets based on silages containing urea is probably associated with blood urea-N elevation.

## CONCLUSION

The inclusion of urea in the corn and WBC silages and formic acid in alfalfa silage caused improve nutritional value especially via altering NDF characteristic during ensiling. Diet containing low uNDF through the urea applied to corn silages increase milk production. Although replacing the corn silage with the WBC silage did not change feed intake, milk yield was higher in the cows fed diets based on corn silage than those fed WBC silage. Overall, reduction of uNDF by the inclusion of urea and formic acid to silages can affect animal performance. However, more research is required to determination of uNDF in all basic silages and feedstuff additives because of its important to estimation potential digestible NDF and ration formulation to achieve better performance.

## Figures and Tables

**Table 1:** Ingredients (% DM) and nutrient composition of dietary treatments

Items	Treatments1)					

	CSAS	CSUAS	CSASF	BSAS	BSUAS	BSASF
Ingredients						
Untreated corn silage	18.0	-	17.9	-	-	-
Urea treated corn silage	-	17.8	-	-	-	-
Untreated barley silage	-	-	-	17.8	-	17.8
Urea treated barley silage	-	-	-	-	17.6	-
Untreated alfalfa silage	14.7	14.7	-	14.9	14.9	-
Formic acid treated alfalfa silage	-	-	14.7	-	-	14.9
Alfalfa hay	7.8	7.8	7.8	7.94	7.9	7.94
Corn grain	16.4	16.2	16.4	16.7	16.7	16.7
Barley grain	17	17	17	17.3	17.3	17.3
Soybean meal 44% CP	13.9	14.5	13.8	14.07	13.8	14.0
Wheat bran	8	8	8	7.11	7.11	7.11
Tallow	1.5	1.5	1.5	1.58	1.58	1.58
Salt	0.10	0.10	0.10	0.12	0.12	0.12
Sodium bicarbonate	0.58	0.58	0.58	0.59	0.59	0.59
Vitamin-mineral2)	0.78	0.78	0.78	0.79	0.79	0.79
Calcium phosphate (Di−)	0.78	0.78	0.78	0.79	0.79	0.79
Magnesium oxide	0.39	0.39	0.39	0.39	0.39	0.39
Chemical composition						
CP (% of DM)	16.2	16.1	16.2	16.2	16.3	16.4
NFC3) (% of DM)	42.6	42.6	42.6	41.7	41.7	41.7
NDF (% of DM)	31.4	31.2	31.3	31.9	31.7	31.6
ADF (% of DM)	18.9	18.9	18.9	18.9	18.9	18.9
Forage NDF (% of DM)	20.8	20.8	20.8	21	21	21
Ether extract (% of DM)	4.1	4.1	4.1	4.3	4.3	4.3
NEL (Mcal/kg of DM)	1.59	1.59	1.59	1.59	1.59	1.59

DM, dry matter; CP, crude protein; NFC, non-fiber carbohydrate; NDF, neutral detergent fiber; ADF, acid detergent fiber; NEL, net energy for lactation.

1) CSAS, untreated corn and alfalfa silages; CS_U_AS, urea-treated corn silage+untreated alfalfa silage; CSAS_F_, untreated corn silage+formic acid-treated alfalfa silage; BSAS, untreated barley and alfalfa silages; BS_U_AS, urea-treated barley silage+untreated alfalfa silage; BSAS_F_, untreated barley silage+formic acid-treated alfalfa silage.

2) Supplied 0.7% Ca, 0.6% P, 36 mg/kg Cu, as well as vitamin A (3,000 IU/kg), vitamin D (800 IU/kg), and vitamin E (6 IU/kg).

3) Calculated by difference 100–(% NDF+% CP+% fat+% ash).

**Table 2:** Particle size distribution of corn and WBC silage used in diets by pen state particle size separator

Items	Corn silage	WBC silage
% DM retained on sieve		
>19.00 mm	25.3	30.3
19.0 to 8.0 mm	57.8	59.5
8.0 to 1.18 mm	15.8	9.2
<1.18 mm	1.1	1
X_gm_ (mm)	12.32	14.05
Standard deviation	2.06	2.11

WBC, whole barley crop; DM, dry matter.

**Table 3:** Chemical composition, pH, aerobic stability, buffering capacity (BC) and *in vitro* undigested NDF at 30 until 240 h of untreated and treated with additives of corn, barley and alfalfa silages

Parameters	Treatments1)						SEM	p-value

	CSC	CSU	BSC	BSU	ASC	ASF		
DM (g/kg)	31.37^a^	30.02^a^	27^b^	26.71^b^	24.6^c^	24.74^c^	0.73	<0.001
pH	3.68^c^	4.10^b^	4.51^ab^	4.71^a^	4.83^a^	4.11^bc^	0.19	0.001
NH_3_-N (mg/dL)	1.86^e^	3.70^b^	2.08^de^	3.94^a^	2.27^cd^	2.35^e^	0.06	<0.001
CP (g/kg)	82.22^f^	101.76^e^	113.61^d^	137.11^c^	172.72^b^	177.73^a^	0.75	<0.001
NDF (g/kg)	558.78^a^	518.57^d^	541.43^b^	529.58^c^	562.42^a^	546.56^b^	3.29	<0.001
ADF (g/kg)	347.83^c^	343.87^c^	365.50^b^	373.73^b^	397.26^a^	401.22^a^	2.85	<0.001
WSC (g/kg)	18.64^b^	17.65^b^	22.40^a^	22.68^a^	8.60^c^	8.49^c^	0.44	<0.001
BC (mE NaOH/100 g)	74.17^c^	74.28^c^	78.56^b^	78.33^b^	118.80^a^	118.66^a^	0.91	<0.001
AS (hours)	33.50^c^	44.66^b^	21^d^	28^c^	70.66^a^	74.66^a^	2.03	<0.001
uNDF_30 h_ (% of NDF)	51.60^e^	48.80^f^	59.21^c^	55.67^d^	67.73^a^	64.42^b^	0.77	<0.001
uNDF_120 h_ (% of NDF)	42.64^d^	41.51^e^	52.19^b^	51.09^c^	54.67^a^	53.89^a^	0.31	<0.001
uNDF_240 h_ (% of NDF)	31.19^c^	29.49^c^	39.42^b^	36.76^b^	46.21^a^	45.33^a^	0.89	<0.001

NDF, neutral detergent fiber; SEM, standard error of the mean; DM, dry matter; CP, crude protein; ADF, acid detergent fiber; WSC, whole barley crop; AS, alfalfa silage; uNDF, undigested neutral detergent fiber.

1) CSC, untreated corn silage; CSU, corn silage treated with urea; BSC, untreated barley silage; BSU, barley silage treated with urea; ASC, untreated alfalfa silage; ASF, alfalfa silage treated with formic acid.

a–f Least squares means within a row with different superscripts differ significantly (p<0.05).

**Table 4:** Feed intake, silage uNDF intake at 30, 120, and 240 h, BW, BCS, body surface temperature, feed efficiency and total tract nutrient digestibility of cows fed diets based on high or low uNDF with silage additives

Parameters	Treatments						SEM	p-value	Contrasts	
	
	Corn silage			WBC silage					High uNDF vs low uNDF	Corn silage vs WBC silage
	
	High uNDF	Low uNDF		High uNDF	Low uNDF					
	CSAS1)	CS_U_AS1)	CSAS_F_1)	BSAS1)	BS_U_AS1)	BSAS_F_1)				
DMI (kg/d)	23.13	22.98	23.26	23.05	23.18	23.24	0.23	0.35	0.44	0.40
Silage uNDF_30h_ intake (kg/d)	2.59^b^	2.32^c^	2.40^c^	2.82^a^	2.51^b^	2.54^b^	0.05	0.04	0.03	0.04
Silage uNDF_120h_ intake (kg/d)	2.09^b^	1.72^c^	1.80^c^	2.31^a^	2.16^b^	2.19^b^	0.04	0.03	0.05	0.03
Silage uNDF_240h_ intake (kg/d)	1.56^c^	1.31^d^	1.49^c^^d^	1.99^a^	1.69^b^	1.89^a^	0.04	0.04	0.03	0.01
BW (kg)	600	597.16	594.16	592.83	593.80	602.66	12.58	0.87	0.86	0.57
BW gain/period (kg/d)	0.32	0.28	0.29	0.27	0.29	0.46	0.10	0.59	0.57	0.33
BCS	3.5	3.1	3.3	3.3	3.3	3.5	0.06	0.53	0.50	0.40
Body temperature (°C)	16.62	21.98	19.01	19.48	18.80	18.97	3.93	0.96	0.76	0.97
Feed efficiency (milk yield/DMI)	1.27^b^	1.35^a^	1.28^b^	1.22^b^	1.24^b^	1.24^b^	0.04	0.04	0.38	0.21
DM digestibility (%)	62.29	68.20	63.90	64.20	65.77	69.44	2.26	0.64	0.53	0.71
CP digestibility (%)	68.29	70.44	71.39	66.88	71.10	60.28	3.33	0.82	0.51	0.79
NDF digestibility (%)	45.26^b^	47.80^a^	47.71^a^	46.08^b^	48.09^a^	47.88^a^	1.18	0.05	0.03	0.44

uNDF, undigested neutral detergent fiber; BW, body weight; BCS, body condition score; WBC, whole barley crop; SEM, standard error of the mean; DMI, dry matter intake; DM, dry matter; CP, crude protein; NDF, neutral detergent fiber.

1) CSAS, untreated corn and alfalfa silages; CS_U_AS, urea-treated corn silage+untreated alfalfa silage; CSAS_F_, untreated corn silage+formic acid-treated alfalfa silage; BSAS, untreated barley and alfalfa silages; BS_U_AS, urea-treated barley silage+untreated alfalfa silage; BSAS_F_, untreated barley silage+formic acid-treated alfalfa silage.

a–c Least squares means within a row with different superscripts differ significantly (p<0.05).

**Table 5:** Milk yield, FCM, ECM and milk composition (percentage or kg/d) of cows fed diets based on high or low uNDF with silage additives

Parameters	Treatments						SEM	p-value	Contrasts	
	
	Corn silage			WBC silage					High uNDF vs low uNDF	Corn silage vs WBC silage
	
	High uNDF	Low uNDF		High uNDF	Low uNDF					
	CSAS1)	CS_U_AS1)	CSAS_F_1)	BSAS1)	BS_U_AS1)	BSAS_F_1)				
Milk yield (kg/d)	29.42^b^	31.07^a^	29.86^ab^	28.25^b^	28.83^b^	28.90^b^	0.76	0.05	0.10	0.07
FCM 3.5% (kg/d)	32.14	33.64	32.53	32.93	32.95	32.51	0.93	0.22	0.32	0.68
ECM (kcal/d)	32.70	34.03	32.71	32.38	33.11	32.72	0.81	0.28	0.23	0.36
Fat (%)	4.07	4.03	4.05	4.52	4.38	4.27	0.17	0.16	0.60	0.03
Fat (kg/d)	1.20	1.23	1.21	1.28	1.26	1.23	0.03	0.12	0.35	0.04
Total protein (%)	3.74	3.62	3.55	3.42	3.70	3.67	0.13	0.36	0.58	0.27
Total protein (kg/d)	1.11	1.13	1.07	0.98	1.06	1.06	0.10	0.42	0.51	0.18
Lactose (%)	5.36	5.30	5.35	5.28	5.38	5.32	0.08	0.61	0.66	0.59
Lactose (kg/d)	1.57	1.60	1.59	1.50	1.55	1.52	0.07	0.38	0.63	0.52
SNF (%)	9.72^a^	9.83^a^	9.51b^c^	9.49^bc^	9.59^b^	9.41^c^	0.10	0.04	0.16	0.05
TS (%)	13.68	13.51	13.26	13.59	13.48	13.34	0.20	0.71	0.31	0.95
Milk urea-N (%)	13.98^c^	18.64^b^	13.98^c^	18.64^b^	23.30^a^	13.98^c^	1.39	0.001	0.05	0.02

FCM, fat-corrected milk; ECM, energy-corrected milk; uNDF, undigested neutral detergent fiber; WBC, whole barley crop; SEM, standard error of the mean; SNF, solid non-fat; TS, total solids.

1) CSAS, untreated corn and alfalfa silages; CS_U_AS, urea-treated corn silage+untreated alfalfa silage; CSAS_F_, untreated corn silage+formic acid-treated alfalfa silage; BSAS, untreated barley and alfalfa silages; BS_U_AS, urea-treated barley silage+untreated alfalfa silage; BSAS_F_, untreated barley silage+formic acid-treated alfalfa silage.

a–c Least squares means within a row with different superscripts differ significantly (p<0.05).

**Table 6:** Ruminal pH value, NH_3_-N and VFA concentrations of cows fed diets based on high or low uNDF with silage additives

Parameters	Treatments						SEM	p-value	Contrasts	
	
	Corn silage			WBC silage					High uNDF vs low uNDF	Corn silage vs WBC silage
	
	High uNDF	Low uNDF		High uNDF	Low uNDF					
			
	CSAS1)	CS_U_AS1)	CSAS_F_1)	BSAS1)	BS_U_AS1)	BSAS_F_1)				
pH	6.27	6.71	6.49	6.47	6.54	6.53	0.31	0.97	0.66	0.98
NH_3_-N (mg/dL)	13.51^d^	14.13^b^	13.30^c^	14.23^b^	14.67^a^	14.04^b^	0.06	<0.001	<0.001	<0.001
Acetate (mM)	52.73	43.88	46.65	62.01	54.94	40.73	6.78	0.32	0.34	0.48
Propionate (mM)	27.96	37.80	27.50	28.84	25.57	30.29	4.95	0.41	0.52	0.26
Butyrate (mM)	6.88	5.98	6.42	5.41	6.64	6.29	1.64	0.58	0.57	0.48
Acetate:propionate	1.89	1.17	1.69	2.15	2.14	1.34	0.51	0.62	0.55	0.31

VFA, volatile fatty acids; uNDF, undigested neutral detergent fiber; WBC, whole barley crop; SEM, standard error of the mean.

1) CSAS, untreated corn and alfalfa silages; CS_U_AS, urea-treated corn silage+untreated alfalfa silage; CSAS_F_, untreated corn silage+formic acid-treated alfalfa silage; BSAS, untreated barley and alfalfa silages; BS_U_AS, urea-treated barley silage+untreated alfalfa silage; BSAS_F_, untreated barley silage+formic acid-treated alfalfa silage.

a–c Least squares means within a row with different superscripts differ significantly (p<0.05).

**Table 7:** Eating, ruminating and water drinking activities of cows fed diets based on high or low uNDF with silage additives

Parameters	Treatments						SEM	p-value	Contrasts	
	
	Corn silage			WBC silage					High uNDF vs low uNDF	Corn silage vs WBC silage
	
	High uNDF	Low uNDF		High uNDF	Low uNDF					
			
	CSAS1)	CSUAS1)	CSASF1)	BSAS1)	BS_U_AS1)	BSAS_F_1)				
Eating (min)	406.66	426.66	426.66	393.33	406.66	405.33	17.63	0.71	0.51	0.21
Eating DM (min)	5.80	6.35	6.29	5.34	5.69	5.50	0.55	0.74	0.64	0.20
Eating NDF (min)	18.60	20.38	20.16	17.08	18.20	17.59	1.78	0.73	0.61	0.19
Rumination (min)	586.66^b^	583.33^b^	580^b^	626.66^a^	613.33^a^	616.66^a^	7.69	0.003	0.42	0.001
Rumination DM (min)	7.93^b^	7.89b	7.40^b^	9.06^a^	8.67^a^	8.70^a^	0.29	0.04	0.52	0.002
Rumination NDF (min)	25.40^b^	25.30^b^	24.82^b^	28.96^a^	27.71^a^	27.80^a^	1.07	0.05	0.50	0.003
Water drinking (min)	36.66	40.00	43.33	46.66	43.33	46.66	5.09	0.71	0.68	0.20
Water DM (min)	1.01	1.12	1.18	1.29	1.20	1.37	0.13	0.77	0.64	0.10
Water NDF (min)	3.23	3.61	3.77	4.14	3.83	4.40	0.43	0.60	0.65	0.10
Total chewing (min)	990	1,010	1,004	1,022	1,019	1,021	24.15	0.72	0.47	0.70
Total chewing DM (min)	14.07	13.94	12.00	14.73	13.63	13.76	0.71	0.41	0.31	0.32
Total chewing NDF (min)	45.11	44.66	38.39	47.08	43.56	43.99	1.98	0.42	0.32	0.34

uNDF, undigested neutral detergent fiber; WBC, whole barley crop; SEM, standard error of the mean; DM, dry matter.

1) CSAS, untreated corn and alfalfa silages; CS_U_AS, urea-treated corn silage+untreated alfalfa silage; CSAS_F_, untreated corn silage+formic acid-treated alfalfa silage; BSAS, untreated barley and alfalfa silages; BS_U_AS, urea-treated barley silage+untreated alfalfa silage; BSAS_F_, untreated barley silage+formic acid-treated alfalfa silage.

a–b Least squares means within a row with different superscripts differ significantly (p<0.05).

**Table 8:** Blood metabolites concentration of cows fed diets based on high or low uNDF with silage additives

Parameters	Treatments						SEM	p-value	Contrasts	
	
	Corn silage			WBC silage					High uNDF vs low uNDF	Corn silage vs WBC silage
	
	High uNDF	Low uNDF		High uNDF	Low uNDF					
			
	CSAS1)	CS_U_AS1)	CSAS_F_1)	BSAS1)	BS_U_AS1)	BSAS_F_1)				
TG (mg/dL)	13.28	21.98	19.01	19.48	18.80	15.33	4.87	0.69	0.53	0.52
CHL (mg/dL)	277.53	283.68	304.78	289.68	282.75	249.30	18.19	0.27	0.77	0.15
Urea-N (mg/dL)	15.73^c^	18.67^ab^	15.60^c^	17.23^b^	19.44^a^	16.06^bc^	1.10	0.03	0.05	0.03
ALT (mg/dL)	20.13	24.38	22.76	23.83	27.73	21.53	1.96	0.28	0.06	0.09
AST (mg/dL)	55.59	68.42	64.77	60.60	59.22	56.37	8.92	0.89	0.65	0.85
Glucose (mg/dL)	56.03	60.28	53.15	55.10	53.28	58.60	2.10	0.32	0.22	0.21

uNDF, undigested neutral detergent fiber; WBC, whole barley crop; SEM, standard error of the mean; TG, triglycerides; CHL, cholesterol; ALT, alanine aminotransferase; AST, apartate aminotransferase.

1) CSAS, untreated corn and alfalfa silages; CS_U_AS, urea-treated corn silage+untreated alfalfa silage; CSAS_F_, untreated corn silage+formic acid-treated alfalfa silage; BSAS, untreated barley and alfalfa silages; BS_U_AS, urea-treated barley silage+untreated alfalfa silage; BSAS_F_, untreated barley silage+formic acid-treated alfalfa silage.

a–c Least squares means within a row with different superscripts differ significantly (p<0.05).
